# Multi-Camera 3D Digital Image Correlation with Pointwise-Optimized Model-Based Stereo Pairing

**DOI:** 10.3390/s25185675

**Published:** 2025-09-11

**Authors:** Wenxiang Qin, Feiyue Wang, Shaopeng Hu, Kohei Shimasaki, Idaku Ishii

**Affiliations:** 1 Graduate School of Advanced Science and Engineering, Hiroshima University, Hiroshima 739-8527, Japan; wenxiang-qin@hiroshima-u.ac.jp (W.Q.); feiyue@hiroshima-u.ac.jp (F.W.); simasaki@hiroshima-u.ac.jp (K.S.); 2 Digital Monozukuri (Manufacturing) Education and Research Center, Hiroshima University, Hiroshima 739-0046, Japan; hsp@hiroshima-u.ac.jp

**Keywords:** 3D dynamic deformation measurement, digital image correlation, multi-camera geometry, automatic camera pairing, 3D model

## Abstract

Dynamic deformation measurement (DDM) is critical across infrastructure and industrial applications. Among various advanced techniques, multi-camera digital image correlation (MC-DIC) stands out due to its ability to achieve wide-range, full-field, and non-contact 3D DDM by pairing camera subsystems. However, existing MC-DIC methods typically rely on inefficient manual pairing or a simplistic strategy that aggregates all visible cameras for measuring specific object regions, leading to camera over-grouping. These limitations often result in cumbersome system setup and ill-measured deformations. To overcome these challenges, we propose a novel MC-DIC method with pointwise-optimized model-based stereo pairing (MPMC-DIC). By automatically evaluating and selecting camera pairs based on five evaluation factors derived from 3D model and calibrated cameras, the proposed method overcomes the over-grouping problem and achieves high-precision DDM of semi-rigid objects. A Ø5 × 5 cm cylinder experiment demonstrated an accuracy of 0.03 mm for both horizontal and depth displacements in the 0.0–5.0 mm range, and validated strong robustness against cluttered backgrounds using a 2 × 4 camera array. Vibration measurement of a 9 × 15 × 16 cm PC speaker operating at 50 Hz, using eight surrounding cameras capturing 1920 × 1080 images at 400 fps, confirmed the proposed method’s capability to perform wide-range dynamic deformation analysis and its robustness against complex object geometries.

## 1. Introduction

Dynamic deformation measurement (DDM) has significant applications such as structural health monitoring [1], industrial maintenance [2], and vehicle refinement [3]. Several contact and non-contact sensors are widely employed for accurate DDM, such as linear variable differential transducers [4], accelerometers [5], fiber optics sensors [6], and laser sensors [7]; yet they all suffer from different problems, like sparse measurements, mass loading, and spatial inconsistency. The rapid advancement in camera resolution has propelled the development of vision-based methods that utilize camera pixels as dense arrays of optical sensors. Vision-based methods are favored for their easy installation, as well as their non-contact, fast, and full-field consistent measurement capabilities. Pixel intensity array-tracking methods, such as digital image correlation (DIC) [8] and template matching [9], are widely adopted for sub-pixel displacement estimation.

Although DIC often requires a speckle pattern applied to the object surface to facilitate intensity matching, it is widely accepted as a non-contact displacement measurement technique, with the advantage of being mass load-free and independent of the tested material or the length scale of interest [10]. DIC was first utilized in the 1980s to monitor aluminum specimens [11], with tracking motion based on photo-consistency between pre- and post-deformation images. Since then, numerous research studies have advanced this technique in terms of refined shape function [12], high-order interpolation [13], enhanced intensity filtering [14], precise and efficient parameter solving [15], and boosted initial guess [16]. To simplify the practical configurations and allow for full-field 3D measurements, 3D-DIC integrated stereo photogrammetry with 2D-DIC was performed in the 1990s [17]. This integration enables 3D DDM using two overlapping images with the support of calibration [18], stereo correspondence [19], and triangulation [20] from stereo photogrammetry. Meanwhile, speckle pattern also enhances calibration based on its rich features and improves measurement precision in the presence of environmental disturbances [21]. Recently, the accuracy of stereo correspondence in 3D-DIC has been significantly enhanced through various strategies, such as image feature description and matching [22], path-guided measurement [23], geometric constrained semi-global matching [24], and model-based projection [25]. Deep learning has further elevated performance by increasing measurement range [26], improving precision, and enabling super-resolution speckle image generation [27]. Based on these advancements, 3D-DIC achieves micrometer-level accuracy by tracking the same patterned subset region across stereo image sequences. However, accurate tracking requires sufficient image overlap between stereo camera views, which inherently restricts the measurable region in 3D-DIC systems [28].

A widely adopted solution for enlarging the measurement region is to use multi-camera systems supported by multi-camera geometry (MCG; also called multi-view geometry). MCG is a technique that is utilized for recovering 3D information from multiple 2D camera images, such as 3D shape measurement [29], 3D DDM [30], 3D position tracking [31], and 3D pose estimation [32]. By capturing images from different locations, MCG strongly compensates for the regions that are difficult to cover by stereo configuration [29], with applications for cultural heritage archival practice, surgical planning, structural health monitoring, crime scene reconstruction, and entertainment [33,34,35,36]. Multi-camera systems are established in two main ways: camera array systems or pseudo-camera systems. Camera array systems place multiple real cameras at different locations, and each camera records an individual view of the object; this configuration ensures high resolution and spatial consistency [37]. Pseudo-camera systems generate virtual cameras by temporal motion, mirror, or a prism, allowing more cost-effective realization [38]. The continued efforts to achieve 3D scene representation [39], calibration [40], stereo correspondence [29], and patchmatch-based depth estimation [29] have strongly advanced its development. On the other hand, multi-camera configurations also make camera grouping (selection) an important issue that is firmly related to measurement accuracy [29]. For accurate 3D shape measurement, several works have modelled it as an optimization problem aiming to achieve optimal camera grouping, based on properties estimated from 2D images, such as visibility, triangulation angle, incident angle, texture, depth, and normal vector [29].

Multi-camera DIC (MC-DIC), which integrates MCG with DIC, is famous for its strong capability for 3D DDM [41]. These methods effectively extend the measurement region by fusing results measured at different object areas [30]. MC-DIC with camera array systems enables wide-range measurements on panoramic and full-region dynamic deformations of column-shaped objects [42] and beam-shaped structures [43], respectively. MC-DIC with pseudo-camera systems supports dual-surface and panoramic measurements while maintaining system compactness and low cost [28]. However, these methods show weakness at camera grouping due to the presence of unavoidable errors in estimated properties [29]. Unlike MCG-based 3D shape measurement, which generally aims at large-scale scenes and shows error tolerance, MC-DIC is sensitive to these errors, as it typically targets small-scale dynamic deformations [28,29]. Nowadays, optimal camera grouping in MC-DIC remains a challenge and few methods have been proposed to target it; most MC-DIC methods typically use pre-paired cameras based on experience-guided manual configuration, which increases the complexity and effort required for system setup [28,30].

Recently, 3D models have been integrated into MC-DIC to represent object surfaces in 3D space for isogeometric analysis [44]; formats include polygon mesh, and non-uniform rational B-spline (NURBS) [45]. The rich prior spatial knowledge from 3D models enables the precise identification of camera visibility and facilitates accurate DDM by grouping visible cameras [46]. This integration has been successfully applied to efficient 3D displacement measurement in aeronautical composite structures [47], with measurement accuracy validated by high consistency with laser scans [48]. However, these methods adopt a simplistic strategy that groups all visible cameras for a given measurement point, which often results in the inclusion of cameras that yield poor measurements [49]. Such over-grouping can introduce ill-measured deformations caused by factors such as object–background discontinuity, self-occlusion, and reflective highlights. Overcoming this issue can significantly improve the robustness against cluttered backgrounds, complex object geometries, and environmental light variations.

To address this gap, we propose a novel MC-DIC method with pointwise-optimized model-based stereo pairing (MPMC-DIC), comprising model-based MC-DIC (MMC-DIC; an extended version of our previous model-based 3D-DIC [25]) and a pointwise-optimized model-based stereo pairing strategy (PMSP). By automatically evaluating multiple cameras and selecting the optimal camera pair for each measurement point on the 3D model based on evaluation factors derived from the 3D model and calibrated cameras, MPMC-DIC overcomes the over-grouping problem and achieves high-precision wide-range 3D DDM of semi-rigid objects. Our main contributions are summarized as follows:

(1) A novel camera pair evaluation metric is proposed for pointwise-optimized model-based stereo pairing in 3D DDM tasks. Since each camera is evaluated individually prior to pair evaluation, the metric can also be applied to assess individual cameras for 2D-DIC.
(2) An MC-DIC method with pointwise-optimized model-based stereo pairing is proposed. To the best of our knowledge, this is the first work dedicated to addressing camera pairing in MC-DIC, enhancing robustness against cluttered backgrounds and complex object geometries.
(3) Experiments were conducted to validate the proposed MPMC-DIC method for 3D DDM, demonstrating micrometer-level accuracy and strong robustness against cluttered backgrounds and complex object geometries.

The paper is organized as follows: Section 2 describes our MPMC-DIC method for 3D displacement estimation based on a pre-measured 3D model. Section 3 validates our MPMC-DIC method of micrometer-level accuracy in measuring a centimeter-sized cylinder and robustness against cluttered backgrounds in comparison with the existing method which groups all visible cameras. Section 4 demonstrates the robustness of our MPMC-DIC method against complex geometries and illustrates its ability to precisely measure the vibrations of objects vibrating at audio frequencies by visualizing detailed vibrational characteristics.

## 2. Method

To achieve effective and efficient wide-range 3D DDM, this study proposes a novel MC-DIC method with pointwise-optimized model-based stereo pairing. Specifically, the proposed method introduces five evaluation factors to overcome the over-grouping problem that typically arises when only visibility is considered. The five evaluation factors derived from the 3D model and multiple cameras, along with their respective functions, are as follows:

- **Visibility**, which determines the availability of cameras for measurement.
- **Subset validity rate**, which reflects the subset’s coverage ratio with measurement object. A lower coverage ratio leads to a greater influence from the background.
- **Subset gradient**, which reflects the depth inclination of measurement object relative to the camera in the subset region, especially depth discontinuities due to self-occlusion.
- **Subset ZNCC similarity** (hereinafter referred to as subset similarity), which reflects the matching confidence of correlated subsets in pre- and post-deformation images.
- **Disparity**, which reflects the angle between a pair of cameras relative to a measurement point. A small disparity often leads to high noise sensitivity, which consequently enlarges the error; a zero disparity disables 3D estimation.

**Figure 1:**
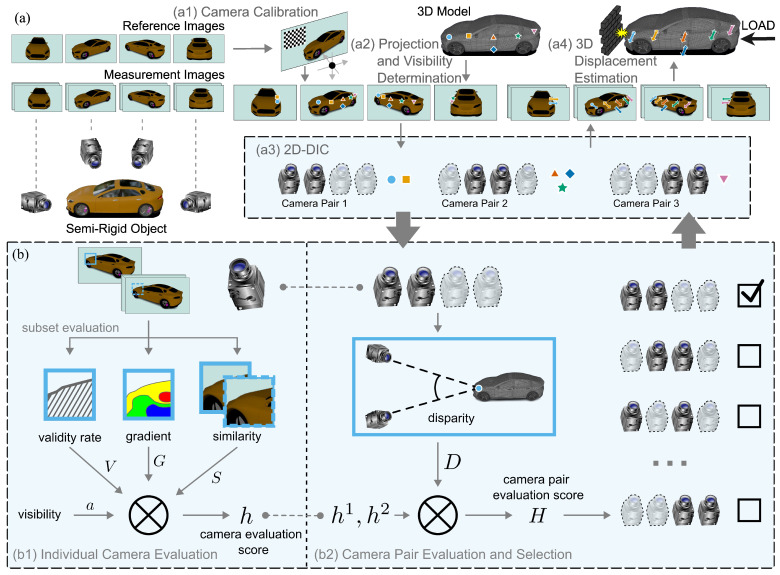
Processing flow of the multi-camera (MC) digital image correlation (DIC) method with the pointwise-optimized model-based stereo pairing (MPMC-DIC) in this study: (**a**) Model-based MC-DIC (MMC-DIC), which involves steps (a1)–(a4). (**b**) Pointwise-optimized model-based stereo pairing strategy (PMSP), which involves steps (b1) and (b2).

Assuming multi-camera image sequences of a semi-rigid object and an associated reference 3D model, the proposed MPMC-DIC, illustrated in Figure 1, comprises MMC-DIC and PMSP. Following pipeline in [25] and integrating multiple cameras and visibility determination, MMC-DIC involves four steps: (a1) Camera calibration to ensure precise measurement. (a2) Projection and visibility determination to identify the spatial relationship between measurement points and cameras. (a3) Two-dimensional-DIC to obtain 2D displacements. (a4) Three-dimensional displacement estimation based on camera pairs selected by PMSP. Note that MMC-DIC can also be applied without PMSP by using manual pairing instead. By leveraging the five evaluation factors, PMSP enables automatic and reliable (b1) Individual camera evaluation; (b2) Camera pair evaluation and selection. This ensures robust and precise wide-range 3D DDM.

MPMC-DIC utilizes multi-view reference images $I_{0}^{c}\left(u,v\right)$ and *K* multi-view measurement images $I^{c}\left(u,v,t\right)$(t=τ,…,Kτ) captured by multiple cameras $C^{c}$, where c=1,…,n. This method assumes that the target object behaves as a semi-rigid body, with a predefined 3D model Ω serving as the reference framework. The 3D model contains *N* measurement points $p_{i}={\left(x_{i},y_{i},z_{i}\right)}^{⊤}$(i=1,…,N) with normal vectors $n_{i}$. Below, we separately outline the detailed algorithm of MMC-DIC in Section 2.1 and PMSP in Section 2.2.

### 2.1. Model-Based MC-DIC (MMC-DIC)

The MMC-DIC algorithm is outlined in detail here, including steps (a1) to (a4), as shown in Figure 1.

(a1) Camera Calibration

Calibration for cameras involves capturing multiple images with known patterns, including the 3D shape of the measurement object. For each camera, intrinsic parameter matrix $M^{c}$ and distortion parameter vector $d^{c}$ are determined.

(a2) Projection and Visibility Determination

The poses of the cameras relative to the 3D model are pre-determined as the extrinsic parameter matrix $R^{c}$ and vector $t^{c}$ as follows:

(1) $$\left(R^{c}\;|\;t^{c}\right)=\mathrm{reg}\left(I_{0}^{c}\left(u,v\right),\Omega \right),$$

where reg(·) registers the 3D model and the reference image to determine extrinsic parameters.

The *N* measurement points on the 3D model are perspectively projected onto the 2D image planes of the cameras. For each camera, the projected points ${\left(u_{i}^{c},v_{i}^{c}\right)}^{⊤}$ are computed using the intrinsic, distortion, and extrinsic parameters:

(2) $$\left(\begin{array}{c} u_{i}^{c}\\ v_{i}^{c} \end{array}\right)={\mathrm{proj}}^{c}\left(p_{i};M^{c},d^{c},R^{c},t^{c}\right),$$

where ${\mathrm{proj}}^{c}\left(\cdot \right)$ denotes the perspective projection.

The visibility of the cameras to each measurement point $a_{i}^{c}\in \left\{0,1\right\}$, as a strict guideline for cameras’ availability for camera pairing, is determined by following conditions:

- The point’s 2D projection is outside the camera image area;
- The point’s normal vector is opposite to the cameras’ orientation;
- The point is occluded by the 3D model surfaces.

If any of the above factors apply, $a_{i}^{c}=0$; otherwise, $a_{i}^{c}=1$. The *c*-th camera is considered for measuring the *i*-th measurement point only when $a_{i}^{c}=1$; this consideration also encompasses its evaluation in PMSP.

(a3) 2D-DIC

Subsets in cameras $\Phi_{i}^{c}$ centered at the *i*-th projected measurement point are set for 2D-DIC. The 2D displacements, ${\left(\Delta u_{i}^{c}\left(t\right),\Delta v_{i}^{c}\left(t\right)\right)}^{⊤}$, of the *i*-th projected measurement point are computed at time *t* for each camera via 2D-DIC by correlating the subset region of reference images in measurement images:

(3) $$\left(\begin{array}{c} {\Delta u}_{i}^{c}\left(t\right)\\ {\Delta v}_{i}^{c}\left(t\right) \end{array}\right)=\mathrm{DIC}\left(I^{c}\left(u,v,t\right),I_{0}^{c}\left(u,v\right),\Phi_{i}^{c}\right),$$

where DIC(·) denotes the 2D-DIC function.

(a4) Three-Dimensional Displacement Estimation

The *N* projected measurement points ${\left(u_{i}^{c},v_{i}^{c}\right)}^{⊤}$ are considered to be displaced to ${\left(u_{i}^{c}\left(t\right),v_{i}^{c}\left(t\right)\right)}^{⊤}$ at time *t* in the 2D images as follows:

(4) $$\left(\begin{array}{c} u_{i}^{c}\left(t\right)\\ v_{i}^{c}\left(t\right) \end{array}\right)=\left(\begin{array}{c} u_{i}^{c}\\ v_{i}^{c} \end{array}\right)+\left(\begin{array}{c} {\Delta u}_{i}^{c}\left(t\right)\\ {\Delta v}_{i}^{c}\left(t\right) \end{array}\right).$$

Camera pairs of the *N* measurement points, $\left\{l_{i}\left(t\right),r_{i}\left(t\right)\right\}$, are determined by PMSP, as outlined in Section 2.2. The 3D positions of the *N* measurement points at time *t*, ${\tilde{p}}_{i}\left(t\right)={\left({\tilde{x}}_{i}\left(t\right),{\tilde{y}}_{i}\left(t\right),{\tilde{z}}_{i}\left(t\right)\right)}^{⊤}$, are estimated via triangulation using their 2D position vectors from selected camera pairs as follows:

(5) $${\tilde{p}}_{i}\left(t\right)=\mathrm{tri}\left(\left\{u_{i}^{c}\left(t\right),v_{i}^{c}\left(t\right)\right\};\left\{l_{i}\left(t\right),r_{i}\left(t\right)\right\},M^{c},d^{c},R^{c},t^{c}\right),$$

where tri(·) denotes the triangulation function. The relative 3D displacement, $\Delta p_{i}\left(t\right)={\left(\Delta x_{i}\left(t\right),\Delta y_{i}\left(t\right),\Delta z_{i}\left(t\right)\right)}^{⊤}$, is computed as the difference between the 3D coordinates of the measurement points:

(6) $$\Delta p_{i}\left(t\right)={\tilde{p}}_{i}\left(t\right)-p_{i}.$$

### 2.2. Pointwise-Optimized Model-Based Stereo Pairing (PMSP)

The PMSP algorithm is outlined in detail here, including steps (b1) and (b2), as shown in Figure 1.

(b1) Individual Camera Evaluation

Depth images $I_{d}^{c}\left(u,v\right)$ are determined for each camera via 2D rendering of 3D model using intrinsic, distortion, and extrinsic parameters. Each pixel of a depth image records the depth distance of its perspectively corresponding 3D model point, or 0 when this pixel is not covered by the 3D model. Mask images $I_{m}^{c}\left(u,v\right)$ define the coverage region of 3D model rendering as follows:

(7) $$I_{m}^{c}\left(u,v\right)=\left\{\begin{array}{cc} 1, & I_{d}^{c}\left(u,v\right)>0,\\ 0, & \mathrm{otherwise}. \end{array}\right.$$

The 3D model rendering coverage ratios in subsets $\Phi_{i}^{c}$ are computed as subset validity rates $V_{i}^{c}$ as follows:

(8) $$V_{i}^{c}=\frac{1}{\left|\Phi_{i}^{c}\right|}\sum_{\left(u,v\right)\in \Phi_{i}^{c}}I_{m}^{c}\left(u,v\right),$$

where $\left|\Phi_{i}^{c}\right|$ computes the pixel number in the subset region.

The inclination degree of the 3D model region projected in the subsets is computed as subset gradients $G_{i}^{c}$ as follows:

(9) $$G_{i}^{c}=\frac{\sum_{\left(u,v\right)\in \Phi_{i}^{c}}I_{m}^{c}\left(u,v\right){\left(\max_{3\times 3}\left(I_{d}^{c}\left(u,v\right)\right)-\min_{3\times 3}\left(I_{d}^{c}\left(u,v\right)\right)\right)}^{2}}{\sum_{\left(u,v\right)\in \Phi_{i}^{c}}I_{m}^{c}\left(u,v\right)},$$

where $\max_{3\times 3}\left(\cdot \right)$ and $\min_{3\times 3}\left(\cdot \right)$ are used to search for the maximum and minimum depth distance, respectively, in a 3 × 3 domain of (u,v) regardless of the pixels uncovered by the 3D model rendering.

The subset similarities, $S_{i}^{c}\left(t\right)$, of the *i*-th projected measurement point are computed at time *t* for each camera via 2D-DIC:

(10) $$S_{i}^{c}\left(t\right)=\mathrm{DIC}\left(I^{c}\left(u,v,t\right),I_{0}^{c}\left(u,v\right),\Phi_{i}^{c}\right).$$

Note that DIC(·) here denotes the same processing as in Equation (3), and subset similarities are computed along with the 2D displacements.

**Figure 2:**
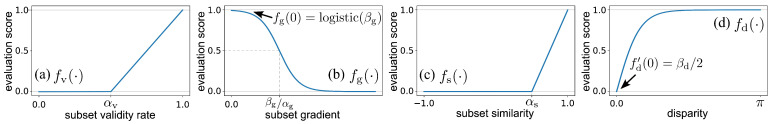
Evaluation functions for (**a**) The subset validity rate. (**b**) The subset gradient. (**c**) The subset similarity. (**d**) The disparity.

Camera evaluation scores at time *t*, $h_{i}^{c}\left(t\right)$, are computed for each measurement point as follows:

(11) $$h_{i}^{c}\left(t\right)=\frac{1}{2}\left[f_{v}\left(V_{i}^{c}\right)f_{g}\left(G_{i}^{c}\right)+f_{s}\left(S_{i}^{c}\left(t\right)\right)\right],$$

where $f_{v}\left(\cdot \right)$ is the evaluation function for the subset validity rate with a parameter $\alpha_{v}$ (Figure 2a) as follows:

(12) $$f_{v}\left(V\right)=\left\{\begin{array}{cc} \frac{V-\alpha_{v}}{1-\alpha_{v}}, & \alpha_{v}\leq V\leq 1,\\ 0, & \mathrm{otherwise}. \end{array}\right.$$

Although the subset validity rate is theoretically defined within the range (0,1], it typically takes values significantly above 0 due to 3D model rendering coverage. Accordingly, its evaluation function $f_{v}\left(\cdot \right)$ is defined as a piecewise function, which employs a monotonically increasing linear function from 0 to 1 over the interval $\left[\alpha_{v},1\right]$, and a constant zero function otherwise, to enhance distinguishability while maintaining the linear relationship. $\alpha_{v}$ is defined in the range [0,1). In practice, it is selected close to the lower bound of the computed subset validity rate distribution, which typically lies around 0.5. $f_{g}\left(\cdot \right)$ is the evaluation function for subset gradient with parameters $\alpha_{g}$ and $\beta_{g}$ (Figure 2b) as follows:

(13) $$f_{g}\left(G\right)=\mathrm{logistic}(\beta_{g}-\alpha_{g}G),$$

(14) $$\mathrm{logistic}(x)=\frac{1}{1+e^{-x}}.$$

To emphasize high evaluation scores for relatively small subset gradients *G* and low scores for relatively large ones, while ensuring a monotonically decreasing trend, and avoiding an abrupt change (e.g., a step function), the subset gradient evaluation function $f_{g}\left(\cdot \right)$ is defined as a flipped and shifted logistic function. Logistic function logistic(·) is a commonly used S-shaped function in machine learning with smooth monotonically values within the range (0,1) [50]. $\mathrm{logistic}(\beta_{g})$ governs the maximum evaluation score at G=0. To provide high and distinguishable evaluation scores for small subset gradients, an empirically acceptable value range for $\beta_{g}$ is [4,8]. $\beta_{g}/\alpha_{g}$ defines the point at which the function decreases to 0.5; in other words, this ratio controls the evaluation of big subset gradients. In practice, $\alpha_{g}$ is selected so that $\beta_{g}/\alpha_{g}$ lies around half of the upper bound of the computed subset gradient distribution. $f_{s}\left(\cdot \right)$ is the evaluation function for subset similarity with a parameter $\alpha_{s}$ (Figure 2c) as follows:

(15) $$\begin{array}{cc} f_{s}\left(S\right)\; & =\left\{\begin{array}{cc} \frac{S-\alpha_{s}}{1-\alpha_{s}}, & \alpha_{s}\leq S\leq 1,\\ 0, & \mathrm{otherwise}. \end{array}\right. \end{array}$$

Similar to $f_{v}\left(\cdot \right)$, the subset similarity evaluation function $f_{s}\left(\cdot \right)$ is defined as a piecewise function, which employs a monotonically increasing linear function from 0 to 1 over the interval $\left[\alpha_{s},1\right]$, and a constant zero function otherwise, to enhance distinguishability, considering that most subset similarity values tend to be concentrated near to 1 in practical applications. $\alpha_{s}$ is defined in the range [−1,1) and, in practice, is selected close to the lower bound of the computed subset similarity distribution.

(b2) Camera Pair Evaluation and Selection

Disparity between the *l*-th and the *r*-th cameras for the *i*-th measurement point, $D_{i}^{lr}$(l,r=1,…,n), is computed using extrinsic parameters as follows:

(16) $$D_{i}^{lr}=\arccos\left(\frac{v_{i}^{l}\cdot v_{i}^{r}}{∥v_{i}^{l}∥∥v_{i}^{r}∥}\right),$$

(17) $$v_{i}^{c}=p_{i}+{R^{c}}^{⊤}t^{c},$$

where $v_{i}^{c}$ denotes the direction vector of the *i*-th measurement point relative to the *c*-th camera.

For a pair containing the *l*-th and *r*-th cameras, the camera pair evaluation score $H_{i}^{lr}\left(t\right)$ is computed as follows:

(18) $$H_{i}^{lr}\left(t\right)=f_{d}\left(D_{i}^{lr}\right)h_{i}^{l}\left(t\right)h_{i}^{r}\left(t\right),$$

where $f_{d}\left(\cdot \right)$ is the evaluation function for disparity with a parameter $\beta_{d}$ (Figure 2d) as follows:

(19) $$f_{d}\left(D\right)=2\cdot \mathrm{logistic}(\beta_{d}D)-1.$$

For a pair of cameras, a big disparity can effectively mitigate the influence of noise, while a small disparity often enlarges the error, and a zero disparity disables 3D estimation. To reduce the evaluation score of a camera pair with small disparity and guarantee a non-zero disparity value, the disparity evaluation function $f_{d}\left(\cdot \right)$ is defined as a scaled and shifted logistic function. Through scaling and shifting, this logistic function ensures the evaluation value is 0 at D=0, and approaches 1 as *D* increases. Compared to monotonically linear function, it offers a steeper growth rate near D=0, followed by a progressively decreasing rate of increase, aligning with the fact that measurement noise sensitivity rises more rapidly as the disparity approaches 0. The parameter $\beta_{d}/2$ controls the maximum rate of increase in the initial phase; in other words, smaller $\beta_{d}$ encourages a stronger tendency toward bigger disparities, while larger $\beta_{d}$ encourages a weaker tendency. $\beta_{d}$ is selected according to the desired encouragement level under the given physical conditions. To ensure effective high and low evaluation scores for camera pairs with big and small disparities, respectively, an empirically acceptable value range for $\beta_{d}$ is [2,20].

The camera pair with the highest evaluation score, $\left\{l_{i}\left(t\right),r_{i}\left(t\right)\right\}$, is selected for each measurement point to estimate the 3D displacement:

(20) $$\left\{l_{i}\left(t\right),r_{i}\left(t\right)\right\}=\underset{l,r}{arg max}\left(H_{i}^{lr}\left(t\right)\right).$$

## 3. Accuracy Verification

### 3.1. Experimental Setting

**Figure 3:**
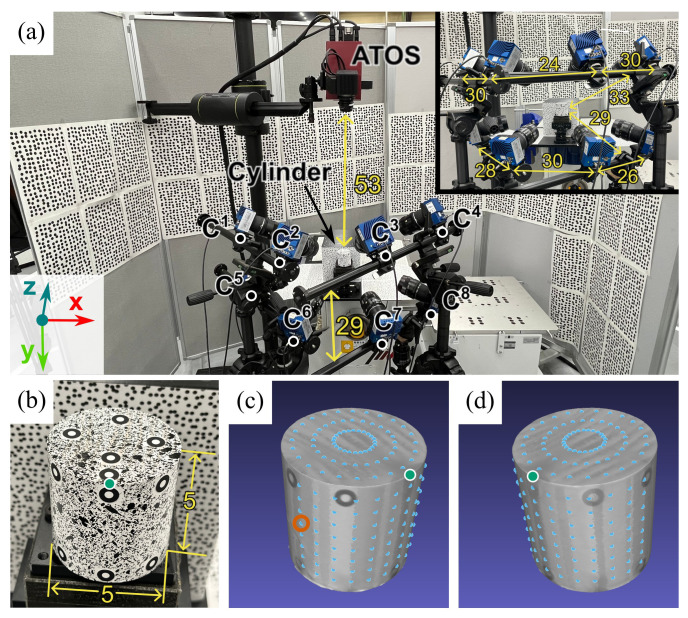
Experimental setup of accuracy verification: (**a**) The experimental scene. (**b**) The cylinder to be observed. (**c**,**d**) A three-dimensional model with 176 measurement points (blue). The green point is located at the upper-front part of the cylinder for alignment across figures. The vermilion circle highlights the measurement point selected for PMSP demonstration (unit: cm).

To assess the robustness of our proposed MPMC-DIC against cluttered backgrounds for wide-range 3D deformation measurement, we validated its accuracy in measuring the rigid displacements of a moving cylinder within a random-speckle background, as shown in Figure 3a, and compared the results with that obtained from the method considering only visibility (hereinafter referred to as the visibility-only method). A Ø5 × 5 cm cylinder (Figure 3b), painted with a random pattern and affixed with 7 mm circular markers as references, was connected to an XYZ stage fixed to the platform. Behind the cylinder, a 15 × 15 × 20 cm cuboid was fixed on the platform with its front surface as a part of the background. Printed speckle patterns were affixed to the platform and background. A high-precision 3D scanner (ATOS Compact Scan, ZEISS, Oberkochen, Germany) with 3-μm uncertainty, positioned 53 cm above the cylinder, measured displacements of five markers on the cylinder’s top surface, with their average as the reference for displacement. The 3D model, reconstructed by the ATOS Compact Scan as shown in Figure 3c,d, included 176 measurement points, consisting of 72 on the top surface (3 circles with 7 mm spacing × 24 in each circle with 15° spacing), and 104 on the front surface (8 semi-circles with 5.5 mm spacing × 13 in each semi-circle with 15° spacing). Eight cameras were arranged in a 2 × 4 curved grid to capture 8-bit Bayer images, covering the cylinder’s top surface and front 180° surface with around 30 cm spacing and measuring distance. Two PCs recorded these Bayer images using CoaXlink Quad CXP-12 frame grabbers (Euresys, Seraing, Belgium) and converted them to grayscale images via the OpenCV function, with each PC connected to four cameras and equipped with an i9-12900K CPU (Intel, Santa Clara, CA, USA), 64 GB RAM, RTX 3090 GPU (NVIDIA, Santa Clara, CA, USA), and Windows 11 Professional. Table 1 shows the optical system configurations.

**Figure 4:**
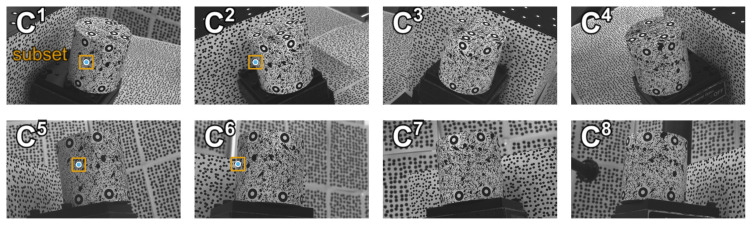
Images captured by eight cameras at the initial position for accuracy verification. $C^{1}$–$C^{8}$ indicate the first to eighth cameras. Blue points and orange squares indicate the projections and 2D-DIC subsets, respectively, of one measurement point as circled in Figure 3c in each visible camera image.

To verify the accuracy of MPMC-DIC, the cylinder was moved along depth (*z*-) and horizontal (*x*-) direction in 11 steps (0.0–5.0 mm, around 0.5 mm per step), with images captured by eight cameras and reference displacement measured using the ATOS Compact Scan at each step. Figure 4 shows images captured at the initial position as reference images. Prior to measurement, cameras were calibrated using checkerboard and registered using circular marker points from the 3D model and 2D reference images. After capturing, recorded images were post-processed to measure displacements for accuracy verification, comparing MPMC-DIC with the visibility-only method. In both methods, the 2D-DIC method in the open-source 3D-DIC tool OpenCorr (version 1.0) [22] was used for 2D displacement measurement, with configurations as shown in Table 2. In MPMC-DIC, evaluation parameters for PMSP were set as shown in Table 3. The mean $\mu_{D}$ and standard deviation $\sigma_{D}$ of the measured displacement $\tilde{D_{i}}$ over 176 measurement points are computed for error analysis. By computing with the reference displacement $D_{\mathrm{ref}}$ and its uncertainty $\sigma_{\mathrm{ref}}$, the mean and standard deviation of error are defined as $\mu_{e}=D_{\mathrm{ref}}-\mu_{D}$ and $\sigma_{e}={\left({\sigma_{\mathrm{ref}}}^{2}+{\sigma_{D}}^{2}\right)}^{1/2}$, respectively, and used as error indices.

### 3.2. Camera Pair Evaluation and Selection

**Figure 5:**
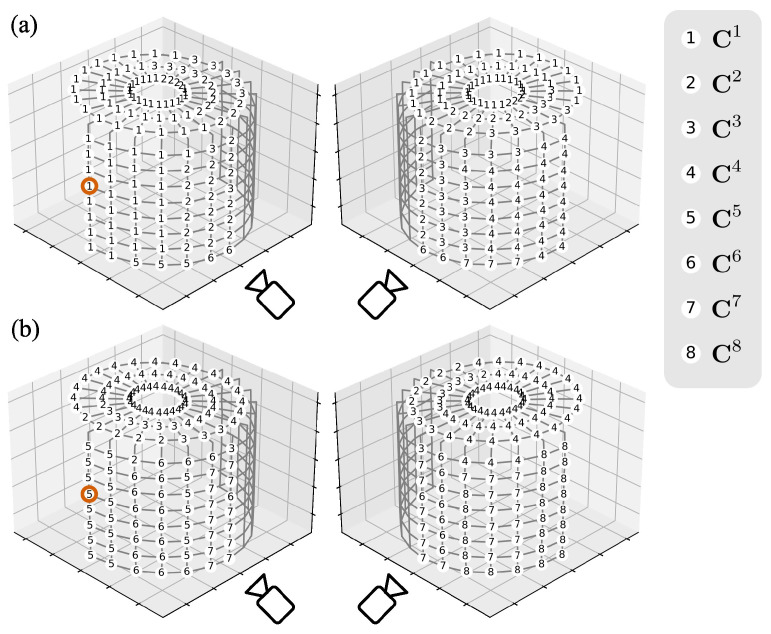
PMSP results for 176 measurement points at 3.0 mm horizontal movement in accuracy verification: (**a**) The first paired camera. (**b**) The second paired camera. The vermilion circle highlights the same measurement point as in Figure 3c selected for PMSP demonstration.

Figure 5 shows the PMSP results at the 3.0 mm horizontal movement. For measurement points on the top surface of the cylinder, paired cameras included the first, second, third, and fourth cameras, while the fifth to eighth cameras were excluded from pairing due to lack of visibility; most of these points selected camera pairs with high disparities for measurement precision, such as the camera pair {1, 4}. All measurement points on the front surface paired two cameras oriented to them; this ensured visibility and prevented large object surface inclination and object–background discontinuity within the subsets for high-quality 2D measurement. Table 4 and Table 5 illustrate the PMSP process for an example measurement point on the front surface of the cylinder, as Figure 3c and Figure 5 circle, at 3.0 mm horizontal movement. In Table 4, each camera was first evaluated individually based on visibility and subset characteristics. As shown in Figure 4, the first, second, fifth, and sixth cameras were visible, while others were not; the subsets from the second and sixth cameras exhibited significant object–background discontinuity, with noticeable background speckles, and higher object surface inclination compared to those from the first and fifth cameras. As a result, the first and fifth cameras achieved high individual evaluation scores *h* exceeding 0.990, whereas the second and sixth cameras obtained lower values due to reduced subset validity rate *V*, similarity *S*, and increased subset gradient *G*; invisible cameras were excluded from the evaluation. In Table 5, the disparity *D* of each camera pair was then evaluated. Most visible camera pairs achieved evaluation scores $f_{d}\left(D\right)$ slightly above 0.9, except for pairs {1, 6} and {2, 5}, which obtained 0.982 and 0.976, respectively. Finally, in the camera pair evaluation stage, the camera pair {1, 5} achieved the highest evaluation score *H* of 0.888, owing to the high individual camera evaluation scores of the first and fifth cameras. This camera pair was therefore selected to measure the example point.

### 3.3. Comparison with Visibility-Only Method

**Figure 6:**
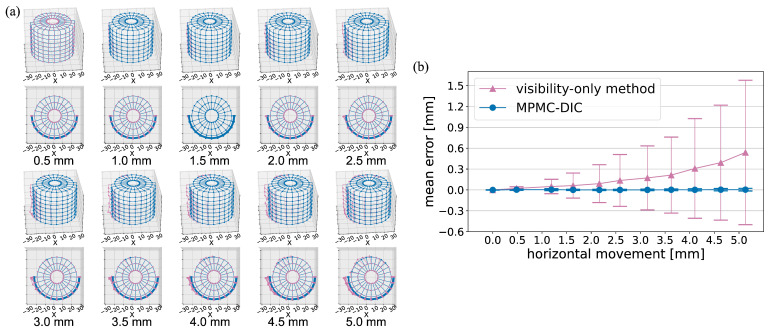
Comparison of displacements measured using MPMC-DIC (blue) and the visibility-only method (red) at different horizontal movements in accuracy verification: (**a**) Displaced 3D position distributions of the 176 measurement points. (**b**) Mean errors $\mu_{e}$ and standard deviations $\sigma_{e}$.

**Figure 7:**
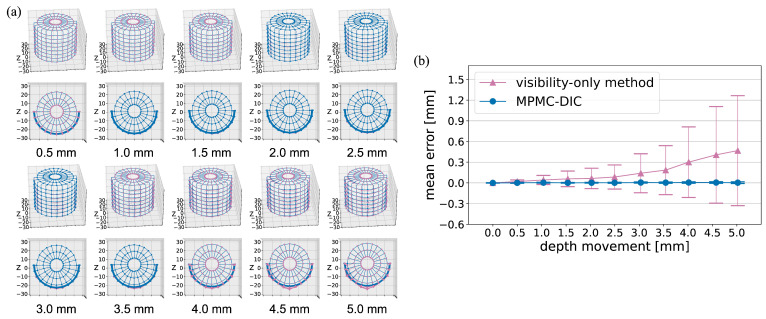
Comparison of displacements measured using MPMC-DIC (blue) and visibility-only method (red) at different depth movements for accuracy verification: (**a**) Displaced 3D position distributions of the 176 measurement points. (**b**) Mean errors $\mu_{e}$ and standard deviations $\sigma_{e}$.

Figure 6 compares MPMC-DIC with the visibility-only method for measuring the horizontal displacements of the cylinder. In Figure 6a, the visibility-only method exhibits increasing deviations with horizontal movements, particularly on the left and top–back sides of the cylinder. These deviations were attributed to over-grouped cameras whose subsets include object–background discontinuity. The presence of background speckles in these subsets led to tracking failures during cylinder movement. In contrast, MPMC-DIC achieved accurate measurements across the entirety of the 0.0–5.0 mm movements by selecting appropriate camera pairs, as demonstrated in Table 4 and Table 5. Quantitative evaluation in Figure 6b shows that the mean error $\mu_{e}$ and standard deviation $\sigma_{e}$ of MPMC-DIC remain below 0.01 mm and 0.02 mm, respectively, whereas the visibility-only method shows rapidly increasing error, reaching a maximum $\mu_{e}$ of 0.54 mm and $\sigma_{e}$ of 1.04 mm at 5.0 mm movement. A similar trend is observed in depth displacement measurement, as illustrated in Figure 7. MPMC-DIC consistently maintains accuracy with $\mu_{e}$ and $\sigma_{e}$ below 0.01 mm and 0.02 mm, respectively, throughout the 0.0–5.0 mm movements, while the visibility-only method exhibits increasing deviations, particularly on the front side due to object–background discontinuity within the subsets from the first, fourth, fifth, and eighth cameras, with a maximum $\mu_{e}$ of 0.47 mm and $\sigma_{e}$ of 0.80 mm.

### 3.4. Verification on Robustness to Digital Speckle Pattern

**Figure 8:**
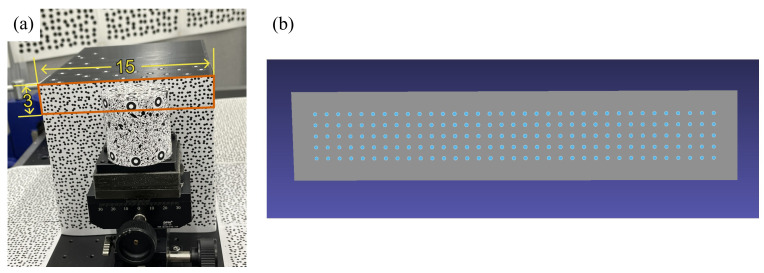
Measurement object in the verification on robustness to digital speckle pattern: (**a**) Planar (vermilion rectangular) object to be observed. (**b**) Its 3D model with 175 evenly assigned (5 × 35) measurement points (blue). The cylinder is considered as background in the measurement (unit: cm).

To verify the robustness of our proposed MPMC-DIC to different speckle types, we also measured the displacements of a planar object with a digital speckle pattern and compared the results with those obtained from the visibility-only method. The 3 × 15 cm planar object was part of the front surface of the cuboid behind the cylinder, as shown in Figure 8a. Its 3D model is shown in Figure 8b, which contains 5 × 35 measurement points evenly distributed at horizontal and vertical spacings of 3.75 mm and 3.95 mm, respectively. The same images, experimental configurations, and error metric described in Section 3.1 were adopted for this measurement and analysis. For the error analysis, the reference displacement was set to zero, since the cuboid remained stationary during image acquisition.

**Figure 9:**
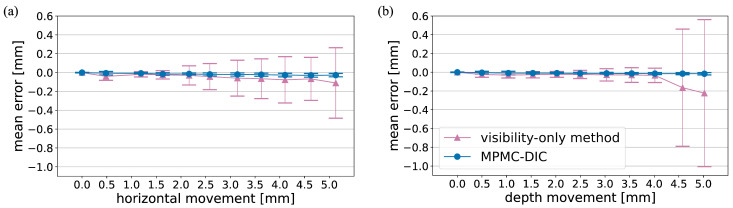
Quantitative comparison of planar object displacements measured using MPMC-DIC (blue) and the visibility-only method (red) in the verification of robustness to digital speckle pattern: mean errors $\mu_{e}$ and standard deviations $\sigma_{e}$ (**a**) at different horizontal cylinder movements; (**b**) at different depth cylinder movements.

Similar to the measurements of cylinder displacements (Figure 6 and Figure 7), MPMC-DIC also outperforms the visibility-only method in measuring planar object displacements (Figure 9), as the visibility-only method was affected by subsets of over-grouped cameras which contained the moving cylinder. Quantitative evaluation at both horizontal and depth cylinder movements shows that the mean error $\mu_{e}$ and standard deviation $\sigma_{e}$ of MPMC-DIC remain below 0.03 mm and 0.02 mm, respectively. In contrast, the visibility-only method exhibits increasing errors, with a maximum $\mu_{e}$ of 0.11 mm and $\sigma_{e}$ of 0.37 mm at 5.0 mm horizontal cylinder movement, and a maximum $\mu_{e}$ of 0.22 mm and $\sigma_{e}$ of 0.78 mm at 5.0 mm depth cylinder movement.

### 3.5. Computational Efficiency Evaluation

To evaluate the computational efficiency of MPMC-DIC relative to the visibility-only method, we recorded the execution time required to measure cylinder displacements under the same conditions and using the same recording PC as described in Section 3.1. The time taken to compute the 3D displacements for one frame of 176 measurement points is summarized in Table 6. When executed sequentially on the CPU, the visibility-only method required 3816.70 s for processing, whereas MPMC-DIC took 30,906.56 s, with camera pairing incurring an additional execution time about seven times that of the visibility-only method. Nevertheless, the PVD and DID procedures dominated the computation for 3D-model-element-wise and pixel-wise operations, making them well-suited for parallel processing. With GPU acceleration, the execution times of PVD and DID were significantly reduced to 167.81 s and 313.47 s, respectively. Consequently, the total execution times of MPMC-DIC and the visibility-only method were reduced to 633.03 s and 312.24 s, respectively, bringing the additional execution time of MPMC-DIC down to nearly the same level as that of the visibility-only method.

In conclusion, unlike the visibility-only method, which loses accuracy with over-grouped cameras, MPMC-DIC maintains accurate measurements by selecting camera pairs with high evaluation scores, requiring only an additional execution time equivalent to that of the visibility-only method to exclude the influence of subsets with object–background discontinuity. These results demonstrate the robustness of our proposed MPMC-DIC against cluttered backgrounds in wide-range 3D deformation measurement.

## 4. Vibration Measurement on PC Speaker

**Figure 10:**
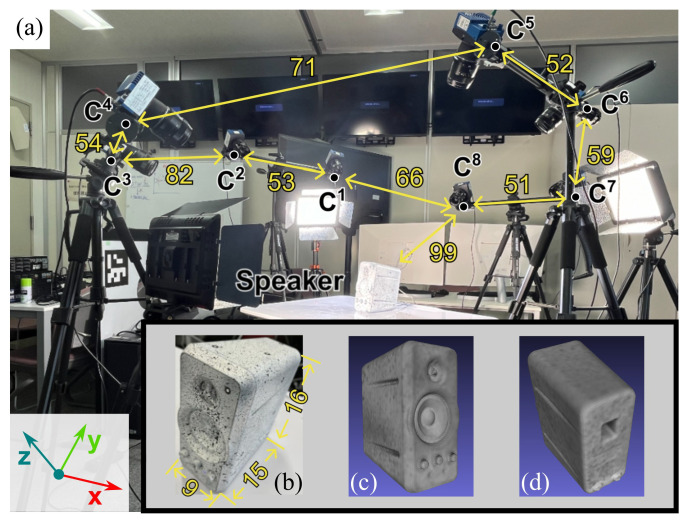
Experimental setup of vibration measurement: (**a**) Experimental scene. (**b**) PC speaker to be observed. (**c**,**d**) Three-dimensional model with 32,055 measurement points across its surface (unit: cm).

**Figure 11:**
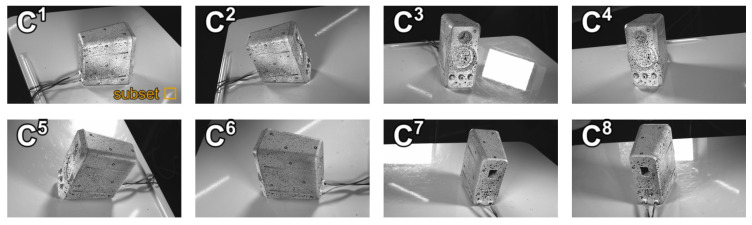
Reference images captured by eight cameras in vibration measurement. $C^{1}$–$C^{8}$ indicate the first to eighth cameras. The orange square in the first image shows the 129 × 129-pixel subset size.

To assess the robustness of our proposed MPMC-DIC against complex object geometries in wide-range 3D DDM, we applied it to measure panoramic vibrations of a 9 × 15 × 16 cm PC speaker featuring a recessed membrane, as shown in Figure 10a, and visually compared it with the visibility-only method. The speaker (Figure 10b) was painted with random pattern and affixed with 7 mm circular markers as references. Its 3D model (Figure 10c,d) was reconstructed by the ATOS Compact Scan, including 32,055 measurement points. The same cameras and PCs as described in Section 3, as well as the same calibration and registration methods, were utilized in the vibration measurement for image capturing, recording, and processing. The eight cameras surrounding the speaker 99 cm away captured 1920 × 1080-pixel reference and measurement images with a 2.3 ms exposure; 400 fps measurement images were captured for 0.5 s when the PC speaker played 50 Hz audio. Figure 11 shows the reference images, where severe self-occlusion can be observed at the membrane part in the second and fifth camera images. Measurement image series were utilized to measure vibration displacements using MPMC-DIC and the visibility-only method based on similar configurations to the accuracy verification: the 2D-DIC method in OpenCorr was performed for 2D measurement using 129 × 129-pixel subsets in both methods, and camera pairs were evaluated and selected using PMSP with the evaluation parameters shown in Table 7. Since the speaker was stable throughout the image capturing process, PMSP was applied solely to the first frame in this experiment, with the subsequent frames using the same camera pair selection. The eight-point moving average component was removed from the measured vibration displacements to suppress the artifacts caused by camera self-motion. The peak-to-peak value of η-direction displacements is calculated as max(Δη(t))−min(Δη(t)), where η=x,y,z. The highest value among three directions is defined as the peak-to-peak vibration value.

**Figure 12:**
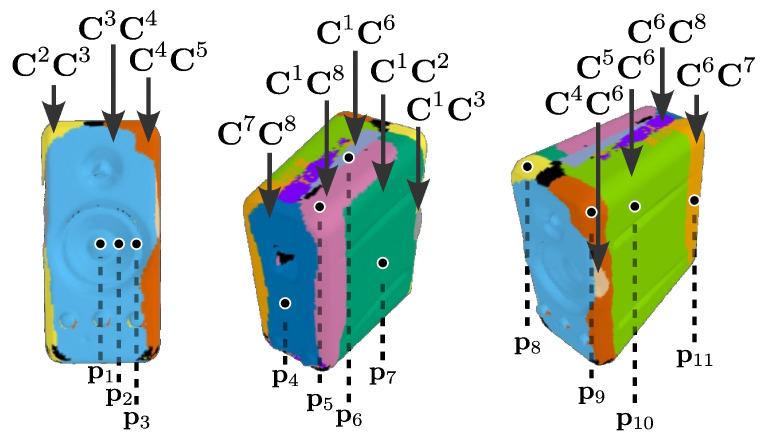
Visualization of the 12 most frequently selected camera pairs with different colors for the first frame processed for vibration measurement. Black regions indicate measurement points which selected other camera pairs or were not measurable due to lack of visibility. In total, 11 of the 32,055 measurement points used for vibration analysis are marked on the 3D model.

Figure 12 visualizes PMSP results. Most measurement points on the front, back, and lateral surfaces paired adjacent cameras which were oriented to these points, as these cameras ensured visibility, along with high evaluation scores in terms of the subset validity rate and gradient. An exception occurred at a few measurement points located on the lateral surfaces close to the membrane region, which selected camera pair {1, 3} or {4, 6}. This resulted from the fact that the second/fifth camera provided low evaluation scores due to severe self-occlusion in the subset region, while other cameras lacked visibility. Regarding measurement points on the top surface, the visibility and similar subset conditions of all cameras allowed them to select a reliable camera pair with higher disparity; for example, some points selected the camera pair {1, 6} or {6, 8}. For measurement points located on the membrane region, the third and fourth cameras were paired, with the first, sixth, seventh, and eighth cameras excluded due to lack of visibility. Although the second and fifth cameras were also visible to part of these measurement points, they were excluded from pairing due to low camera pair evaluation scores resulting from severe self-occlusion, as illustrated in Figure 11.

**Figure 13:**
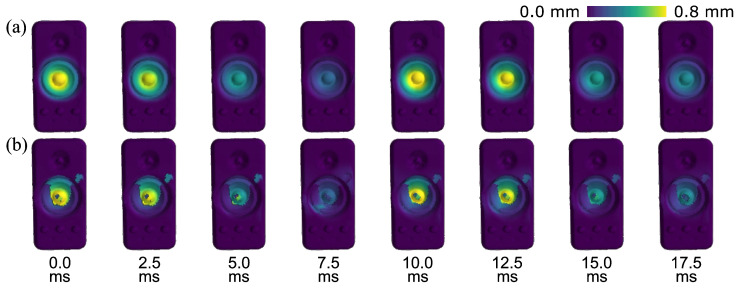
Time-varying 3D shapes of the speaker’s front surface over 20 ms (one period of 50 Hz audio), placing emphasis on the membrane vibration measured by different methods: (**a**) MPMC-DIC; (**b**) visibility-only method. Deformations are visually magnified 10 times in geometry, and enhanced by colormap based on the actual displacement magnitude (∥Δp(t)∥).

**Figure 14:**
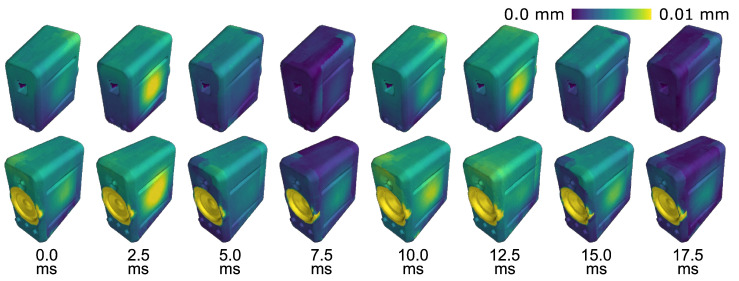
Time-varying 3D shapes of the speaker over 20 ms (one period of 50 Hz audio), placing emphasis on the housing vibration measured by MPMC-DIC. Deformations are visually magnified 10 times in geometry, and enhanced by colormap based on the actual displacement magnitude (∥Δp(t)∥).

Based on the PMSP, our MPMC-DIC achieved accurate 3D vibration measurements, as shown in Figure 13a, revealing a circular vibration distribution centered on the membrane. The vibration amplitude reaches its peak at the center and gradually diminishes toward the edges. In contrast, the visibility-only method, as shown in Figure 13b, exhibits substantial deviations due to over-grouping of all visible cameras. Ill-measured deformations from the second and fifth cameras led to remarkable errors. As for the measurements on the speaker housing, Figure 14 presents the periodic vibration measured using MPMC-DIC. Circular vibration distributions excited by the 50 Hz audio are observed at the left and right lateral surfaces. Due to measurement biases among cameras, minor spatial discontinuities in the measured deformations are observed along the boundary where pairing alters, like the transition region between the top and left lateral surfaces at 7.5 ms. Nevertheless, future work is expected to mitigate these discontinuities by grouping a larger number of cameras and adopting weighted triangulation.

**Figure 15:**
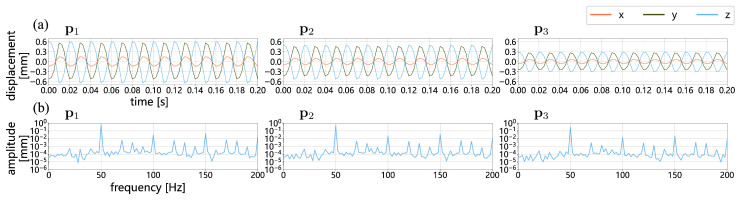
Three-dimensional vibrations measured at three measurement points at the membrane of the speaker ($p_{1-3}$ as illustrated in Figure 12) using MPMC-DIC: (**a**) Three-dimensional displacements over 0.2 s; (**b**) frequency–amplitude spectra derived from 0.5-s *z*-displacements, where a prominent peak at 50 Hz, as well as harmonic peaks at 100 and 150 Hz are identified. $p_{1}$ and $p_{3}$ are located at the center and edge of the membrane, respectively, and $p_{2}$ is at the middle of them.

**Figure 16:**
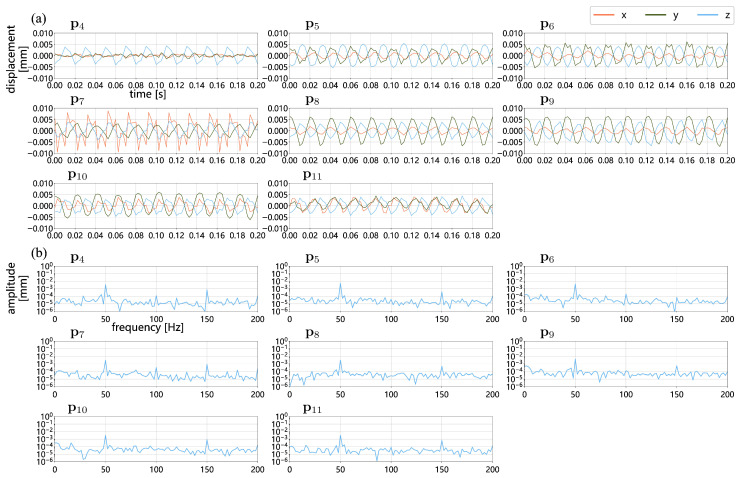
Three-dimensional vibrations measured at eight measurement points on the back, top, and lateral surfaces of the speaker ($p_{4-11}$ as illustrated in Figure 12) using MPMC-DIC: (**a**) three-dimensional displacements over 0.2 s; (**b**) frequency–amplitude spectra derived from 0.5-s *z*-displacements, where a prominent peak at 50 Hz, as well as harmonic peaks at 100 and 150 Hz are identified.

Figure 15 presents the 3D vibrations of three points ($p_{1}$, $p_{2}$, and $p_{3}$) located on the speaker membrane. Corresponding to the distribution shown in Figure 13a, these points exhibit gradually decreasing peak-to-peak vibration values of 1.252, 1.032, and 0.598 mm, respectively. Their *z*-direction frequency amplitude spectra reveal a prominent peak at 50 Hz, aligned with the speaker’s operating frequency, as well as harmonic peaks at 100 and 150 Hz. Figure 16 shows the 3D vibrations of eight points located on the speaker housing, with respective peak-to-peak vibration values of 0.009, 0.011, 0.012, 0.018, 0.013, 0.013, 0.012, and 0.009 mm. Although the vibration amplitudes of these points are significantly smaller than those on the membrane, their frequency responses exhibit a similar pattern, with maximum amplitudes at 50 Hz and harmonic peaks at 100 and 150 Hz. Across all 11 points, the frequency amplitude spectra reveal that the amplitudes at 150 Hz are slightly or significantly greater than those at 100 Hz. This indicates the presence of a mechanical resonance in the PC speaker structure at 150 Hz, in addition to the harmonic components.

These results demonstrate that our proposed MPMC-DIC enables accurate wide-range 3D DDM by selecting camera pairs with high evaluation scores, effectively handling the self-occlusions caused by complex geometries and minimizing the deviations observed in the visibility-only method, thereby confirming its robustness.

## 5. Conclusions

In this study, we propose a novel MPMC-DIC method for wide-range 3D DDM of semi-rigid objects. The proposed method enables automatic camera pair evaluation and selection, filtering out cameras which potentially provide ill-measured deformations, thus ensuring accurate measurements. Experiments which measure cylinder rigid displacements and PC speaker vibrations validate its accuracy, along with robustness against cluttered backgrounds and complex object geometries in comparison with the visibility-only method. Visual and quantitative evaluations demonstrate large deviations with a maximum mean error of 0.54 mm for the visibility-only method, whereas MPMC-DIC maintains a mean error below 0.03 mm with an additional execution time approximately equivalent to that of the visibility-only method. Minor spatial discontinuities are observed in the measured deformations along boundaries where camera pairing changes, due to biases among cameras; this issue is expected to be mitigated in future work by grouping multiple cameras. Although this has yet to be proven, MPMC-DIC may enhance robustness in more practical situations, such as environments with light variations, based on its capability to pair reliable cameras.

Being a newly proposed technique, MPMC-DIC demonstrates considerable promise to applications like in situ monitoring with uncontrollable backgrounds, assessment of products with complex geometries, and measurement in environments with reflective highlights. Our work will further demonstrate its practical performance and continue to extend its measurement capabilities, including local strain measurement. Deeper investigations in the future will be strongly beneficial to develop this method into a highly effective and impactful tool, particularly with regard to the following aspects:

- Evaluation factors and functions: The five evaluation factors were selected based on experience, and evaluation functions were designed based on the requirement for specific factor evaluation, but they have not been comprehensively evaluated to be strictly limited as described in this paper. More forms of evaluation factors or functions are encouraged to be applied in expectation of a better distinguishment between reliable and ill-conditioned camera pairs.
- Automatic parameter determination: The evaluation parameters for PMSP in the study were artificially assigned. Future research on automatic parameter determination can further simplify the system setup, e.g., by integrating with machine learning or deep learning.
- Multiple-camera grouping: Although cameras were paired into stereo subsystems in this study, a subsystem is able to involve more cameras, and this extension is believed to enable higher-accuracy 3D DDM with weighted triangulation.
- Active camera positioning: Our evaluation metric was applied for camera pairing based on static camera positions. In the future, it is also possible that it could be utilized for active camera positioning, or for both of these at the same time.

## Figures and Tables

**Table 1:** Optical system configurations.

Camera	Eosens 2.0CXP2 HFR camera (Mikrotron, Unterschleissheim, Germany)
Resolution	1920 × 1080 pixels
Pixel size	10 μm
Lens	CREATOR F2 lens (Zhong Yi Optics, Shenyang, China)
Focal length	35 mm
Aperture	f/16
Stand-off distance (SOD) ^1^	273.03 mm
Stereo angle ^2^	43°
Field-of-view (FOV) ^3^	149.78 × 84.25 mm
Image scale ^3^	12.82 pixel/mm

^1^ The SOD was calculated as the mean distance of the eight cameras from the cylinder. ^2^ The stereo angle was calculated as the mean angle between adjacent cameras. ^3^ The FOV and image scale were theoretically derived based on the SOD. The actual FOV is presented in Figure 4, and the actual image scale may vary slightly depending on the measurement position.

**Table 2:** Two-dimensional-DIC configurations.

Subset size	161 × 161 pixels
Initial guess	fast Fourier transformation accelerated cross-correlation (FFTCC)
Non-linear optimization	inverse compositional Gauss–Newton algorithm (ICGN)
Subset shape function	first-order shape function
Convergence criterion	0.001 pixels
Stop condition	10 iteration steps
Correlation criterion	zero-mean normalized cross-correlation (ZNCC)
Interpolation	bi-cubic B-spline interpolation

**Table 3:** PMSP evaluation parameters in accuracy verification.

$\alpha_{v}$	$\alpha_{g}$	$\beta_{g}$	$\alpha_{s}$	$\beta_{d}$
0.5	12.5	5.0	0.5	4.0

**Table 4:** PMSP flow (individual camera evaluation) in accuracy verification.

	*a*	*V*	*G*	*S*	$f_{v}\left(V\right)$	$f_{g}\left(G\right)$	$f_{s}\left(S\right)$	*h*
$C^{1}$	**1**	**1.000**	**0.025**	**0.996**	**1.000**	**0.991**	**0.993**	**0.992**
$C^{2}$	1	0.592	0.430	0.899	0.184	0.406	0.799	0.437
$C^{5}$	**1**	**1.000**	**0.005**	**0.994**	**1.000**	**0.993**	**0.987**	**0.990**
$C^{6}$	1	0.604	0.190	0.647	0.207	0.932	0.294	0.244
others	0	*	*	*	*	*	*	*

Values shown in bold indicate the results associated with the selected camera pair. Evaluations for invisible cameras are excluded and replaced with labels “*”.

**Table 5:** PMSP flow (camera pair evaluation and selection) in accuracy verification.

	$f_{d}\left(D\right)$	$C^{1}$	$C^{2}$	$C^{5}$	$C^{6}$	Others	Camera Group	*H*
*D*								
$C^{1}$		/	0.914	**0.904**	0.982	*	$C^{1}C^{2}$	0.396
$C^{2}$		0.775	/	0.976	0.908	*	$C^{1}C^{5}$	**0.888**
$C^{5}$		**0.747**	1.107	/	0.936	*	$C^{1}C^{6}$	0.237
$C^{6}$		1.173	0.758	0.853	/	*	$C^{2}C^{5}$	0.422
others		*	*	*	*	/	$C^{2}C^{6}$	0.097
							$C^{5}C^{6}$	0.226
							others	*

Values shown in bold indicate the results associated with the selected camera pair. Evaluations for invisible cameras are excluded and replaced with labels “*”.

**Table 6:** Computational time of MPMC-DIC and visibility-only method.

		Time [s]							
Method		Calibration	PVD ^1^	2D-DIC	3D-DE ^2^	DID ^3^	ICE ^4^	CPES ^5^	Total
visibility-only method	CPU ^6^	135.89	3671.80	9.01	0.00	/	/	/	03816.70
	GPU ^7^	-	0,167.34	-	-	/	/	/	00,312.24
MPMC-DIC	CPU ^6^	135.89	3677.05	8.88	0.00	27,077.75	6.87	0.11	30,906.56
	GPU ^7^	-	0,167.81	-	-	00,313.47	-	-	00,633.03

^1^ PVD refers to projection and visibility determination. ^2^ Three-dimensional-DE refers to 3D displacement estimation. ^3^ DID refers to depth image determination. ^4^ ICE refers to individual camera evaluation. ^5^ CPES refers to camera pair evaluation and selection. ^6^ CPU indicates that execution times were recorded using sequential procedures on the CPU for algorithm implementation without multithreading. ^7^ GPU indicates that the execution times of PVD and DID were recorded using parallel procedures on the GPU for algorithm implementation with 32,768 threads. Other execution time with labels “-” remain the same as those recorded while using CPU.

**Table 7:** PMSP evaluation parameters in vibration measurement.

$\alpha_{v}$	$\alpha_{g}$	$\beta_{g}$	$\alpha_{s}$	$\beta_{d}$
0.5	0.5	5.0	0.6	12.0

## Data Availability

Data are contained within the article.
